# Indications and Outcomes of Video Capsule Endoscopy in Sub-Saharan Africa: A 5-Year Single-Center Experience in Nairobi, Kenya

**DOI:** 10.1155/jotm/6495299

**Published:** 2025-07-23

**Authors:** Werimo Pascal Kuka, Gloria Wangechi Mugo, Emmanuel Benge Oluoch, Eric Mwenda Murunga, Nelson O. Onyango, Kofi Clarke

**Affiliations:** ^1^Gastro and Liver Centre, Nairobi, Kenya; ^2^Department of Medicine, Aga Khan University Hospital, Nairobi, Kenya; ^3^Department of Medicine, Hospital of the University of Pennsylvania, Philadelphia, Pennsylvania, USA; ^4^Division of Gastroenterology and Hepatology, Department of Medicine, Penn State Health Milton S. Hershey Medical Center, Hershey, Pennsylvania, USA

**Keywords:** Africa, angiodysplasia, endoscopy, gastrointestinal bleed, helminths, intestinal tuberculosis, Kenya, video capsule endoscopy

## Abstract

**Background:** Utilization of video capsule endoscopy (VCE) for the evaluation of small bowel disease is limited in Africa. The predominant causes of gastrointestinal disease in this region are infectious, but the prevalence of inflammatory bowel disease and malignancies is rising. We sought to evaluate the indications and outcomes of VCE in sub-Saharan Africa.

**Methods:** We conducted a retrospective study at an outpatient gastroenterology center in Nairobi, Kenya. Data collected included demographics of the study population, procedure indication, prior investigations, findings, and complications of VCE. Descriptive statistics were used to analyze the results, and statistical analysis of association was performed using Fischer's exact test; a *p* value of less than 0.05 was considered significant.

**Results:** A total of 72 patients underwent VCE from January 2017 to April 2022. The mean age was 59.1 years (range: 15–91); 61.7% were males. A total of 97.2% of the patients had a preceding upper and lower endoscopy. The indications were obscure gastrointestinal bleeding (GIB) in 51.4%, anemia (15.5%), abdominal pain (25.4%), altered bowel habits (23.9%), weight loss (9.9%), and suspected Crohn's disease (7.0%). The overall diagnostic yield was 77.1% and included angiodysplasia (14%), duodenitis (10.7%), mass/polyps (8.9%), suspected intestinal tuberculosis (7.1%), and helminths (2.8%). Angiodysplasia was the most common cause of GIB, accounting for 35.3% and 35.0% of occult and overt bleeding, respectively. Capsule retention occurred in 2 patients (2.8%).

**Conclusions:** There are similarities in indications and preceding workup in our cohort compared to existing literature from Western and Asian countries. Infectious causes of GIB were unique to our study.

## 1. Introduction

Video capsule endoscopy (VCE) is used to evaluate small bowel diseases, an area of the bowel not fully accessible through conventional upper and lower gastrointestinal (GI) endoscopy. Since its introduction in 2000, VCE has undergone technological advancements, including the availability of different models, improvement in image quality, and the use of assisted devices and manipulated locomotion for better visualization [1–3]. More recently, the potential role of artificial intelligence (AI) for lesion detection and reduction of physician burden in reading VCE frames is being recognized [4, 5]. Despite its increasing availability in many regions, data regarding the access and utilization of VCE in sub-Saharan Africa (SSA) is limited.

A common indication for VCE is obscure gastrointestinal bleeding (GIB)—in which it has a better diagnostic yield than other modalities, such as push enteroscopy and traditional radiological techniques [6–8]. Other indications include anemia, Crohn's disease, small bowel tumors, unexplained abdominal pain, and malabsorption syndromes including celiac disease [1]. The epidemiology of these conditions varies in different regions of the world. For instance, the etiology of iron deficiency anemia in SSA includes chronic GI blood loss from helminths. Although there are noninvasive and affordable tests for worms, VCE has detected helminths in reports from other regions [9, 10]. In the evaluation of suspected Crohn's disease, international guidelines recommend using VCE as the initial diagnostic tool for suspected disease after negative ileocolonoscopy findings [11]. Previous reports indicate a low incidence of inflammatory bowel disease in SSA. Recent data suggest an increasing prevalence in this region [12].

Capsule retention is an infrequent but serious complication of VCE occurring in about 1%-2% of the procedures, rates that are higher in stenotic inflammatory lesions and tumors, and requires endoscopic, laparoscopic, or surgical retrieval [13–15]. Other complications are the inability to swallow the capsule and delayed gastrointestinal tract (GIT) passage manifesting as delayed transit through the pylorus or failure to reach the ileocecal valve. Technical problems can include short device battery life and download failures from the recorder [15].

Few centers in SSA utilize VCE as a diagnostic tool. The region traditionally has had a huge burden of infectious diseases but is also witnessing an increase in noncommunicable diseases including malignancies and inflammatory bowel diseases. We reviewed the indications, findings, and complications of VCE at a facility in Kenya.

## 2. Methods

### 2.1. Study Design

We performed a retrospective review of the medical records of all 72 patients who underwent capsule endoscopies at Gastro and Liver Center from January 2017 to April 2022, in Nairobi, Kenya. We obtained medical histories and reviewed electronic and paper medical records. We collected demographic data, indications for capsule endoscopy, prior workup, VCE findings, and complications. The documented procedures before VCE included upper and lower endoscopies, computed tomography (CT) scans, barium studies, angiographies, and push enteroscopy. Study personnel (W.P.K., G.W.M., E.B.O., and N.O.O.) collected the data by reviewing records and completed data entry into a secure online database, Research Electronic Data Capture (REDCap) hosted at the Aga Khan University, Nairobi. The review was retrospective and noninterventional and as such was considered an exempt study. This study was approved by the Nairobi Hospital Institutional Scientific Ethics and Research Committee (approval no. TNH/DMSR/ISERC/RP/005/24).

We classified indications as occult GIB, overt GIB, anemia, altered bowel habits, abdominal pain, suspected Crohn's disease, suspected intestinal tuberculosis (TB), weight loss, and miscellaneous. Overt/gross GIB was defined as a history of hematochezia, melena, or hematemesis. We defined occult GIB as a positive fecal occult blood test (immunochemical or guaiac) without gross bleeding; we then characterized altered bowel habits as either diarrhea or constipation without a known cause. Anemia was defined as hemoglobin below the lower limit of the normal range but without evidence of overt or occult bleeding. Indication of abdominal pain was documented if there was no clear etiology after a prior workup. Patients with self-reported weight loss or documented reduction in weight on medical records were also categorized as such and all other indications recorded under the miscellaneous. Patients with more than one indication for VCE had entries for each of the distinct indications.

### 2.2. VCE System and Protocol

We used the Capsocam SV (Capso-Vision, USA) video capsule from January 2017 to June 2018 and the PillCam SB3 (Medtronic, USA) video capsule from June 2018 to April 2022. Both systems comprised the capsule endoscope, sensors attached to a data recorder, and computer software for image review.

All patients received bowel preparation consisting of a low residue diet for 24 h, 2 L of polyethylene glycol-based electrolyte solution 8 h before, and fasting for 6 h. The progress of the capsule was checked on the recorder after 1 hour, 4 hours, and 8 hours. Prokinetic therapy (10 mg domperidone) was administered if the capsule stayed in the stomach for more than one hour. The patients did not have their first meal until at least 5 hours after ingesting the capsule and could continue normal physical activities with progress monitoring via the portable recorder.

After the data had been transferred from the recorders, the capsule images were reviewed using CapsoView software (January 2017–June 2018) and PillCam Rapid version 8.00 Software (June 2018–April 2022) and verified by the gastroenterologist (EM), with aid from the associate doctor. The authors then reviewed the pertinent images based on the report provided by the gastroenterologist.

We classified the abnormal findings on VCE as angiodysplasia, mass/polyps, gastritis, duodenitis, ileitis, nonsteroidal analgesic (NSAID enteropathy), suspected Crohn's disease, suspected intestinal TB, helminths, blood without definite lesion, and miscellaneous. The latter comprised findings that did not fit the categories listed above, and findings were considered clinically significant if they explained the indication for VCE. The presence of bleeding or stigmata of recent bleeding was considered a positive finding. Patients were considered to have suspected Crohn's disease or intestinal TB if lesions found on VCE were consistent with clinical suspicion, from referral notes, gastroenterologist E.M.M.'s initial assessment, and previous investigations.

A complete exam was defined as the capsule passing through the ileocecal valve or into the colon during the recording. Retention of VCE was defined as the capsule remaining in the digestive tract for more than 72 h, with confirmatory x-ray imaging or requiring intervention for its passage or removal. Reasons for retention and any procedures required for retrieval were recorded.

### 2.3. Statistical Analysis

Analysis and summary of the results were done using descriptive statistics and the commercially available Statistical Package for Social Sciences (SPSS) Version 25. Statistical analysis of association was performed using Fischer's exact test and a *p* value of less than 0.05 was considered significant. The diagnostic yield, frequency of indications, and outcomes of the VCE were calculated. In addition, we evaluated various demographic and clinical characteristics to determine which factors were associated with positive outcomes.

## 3. Results

Data from a total of 72 unique video capsule endoscopies from January 2017 to April 2022 were collected. Mean age of study patients was 59.1 years (range: 15–91), with 64.7 years for men and 50.2 years for women. Males constituted 61.7% (44/72) of the patients. About two thirds of the study population (50/72) were of African race. Persons of Asian descent and Caucasians accounted for 20.8% and 9.7% of the population, respectively.

Most patients underwent upper and lower endoscopy before the capsule procedure. Other investigations done included CT abdomen (29/72), angiography (4/72), barium swallow (2/72), and only 1 patient had undergone a push enteroscopy. The details are shown in Table 1.

The most common indications for capsule endoscopy were both occult and overt GIB, accounting for 23.6% (17/72) and 27.8% (20/72), respectively. Overall, GIB was the most common indication, constituting 51.4% of the patients who had capsule endoscopy performed. Other indications included abdominal pain (18/72, 25.0%), anemia (11/72, 15.3%), altered bowel habits (17/72, 23.6%), weight loss (7/72, 9.7%), and suspected Crohn's disease (5/72, 6.9%). Miscellaneous indications, each accounting for a single participant, were elevated chromogranin A levels, and surveillance following gastrointestinal stromal tumor (GIST) treatment.

**Figure 1:**
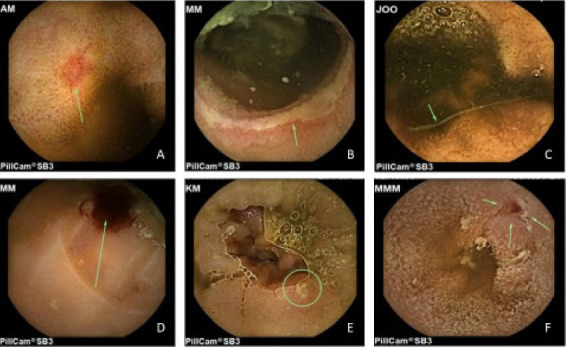
VCE findings: (A) angiodysplasia, (B) longitudinal ulcer, (C) worms, (D) active bleeding, (E) ileal ulcer, and (F) ileal ulcer with stigmata of recent bleeding.

A complete examination was achieved in 65/72 (90.2%), and the overall diagnostic yield was 77.8% (56/72). The reasons for incomplete examination included gastroparesis, intestinal dysmotility, and poor visibility. The most frequent intestinal findings were angiodysplasia and duodenitis in 19.4% (14/72) and 8.3% (6/72) of the procedures, respectively. Gastritis was the most common nonsmall bowel finding, detected in 26.4% (19/72). The frequency of the findings is summarized in Table 2, whereas select images of findings are depicted in Figure 1. There were no statistically significant differences in participants younger than 60 years and those older than 60 years (Supporting Figure 1).

Most common cause for GIB was angiodysplasia, found in 35.3% and 35.0% of patients with occult and overt bleeding, respectively. Overall, 80% (16/20) of the patients with overt bleeding and 70.6 (12/17) of the patients with occult bleeding had an abnormality detected.  Table 3 summarizes the small bowel findings based on indications for capsule endoscopy. Amongst the seven patients with abdominal pain as the sole indication for VCE, only 2 (28.6%) had abnormal findings of duodenitis and colon ulcers (Supporting Table 1).

Capsule retention occurred in 2/72 (2.8%) of the participants. In one patient, the capsule was retained in the stomach for more than 6 hours and required endoscopic manipulation for its passage into the duodenum. The second patient had nonpassage of the capsule after 72 h of ingestion. The patient had no complaints suggestive of intestinal obstruction. A subsequent CT abdomen performed a week after the procedure showed no capsule, suggestive of spontaneous passage.

## 4. Discussion

In this retrospective study, we evaluated the use of capsule endoscopy, an expensive but limited resource, in a developing country. We assessed the indications, preceding investigations, and outcomes of VCE at a single center in Nairobi, Kenya. Data regarding the use of the procedure in SSA are limited and to our knowledge, this is the first study from this region that documents the experience of VCE use in clinical practice.

Abnormal findings which were noted in 77.8% of the procedures were higher than the yield in previous reports which ranged between 47% and 60% and support the notion of a high diagnostic yield of VCE [14, 16, 17]. The detection rates for overt and occult GIB, the most common indications for VCE, were 80% and 70.6%, respectively. The yield in overt GIB is significantly higher than occult GIB in multiple reports [6, 14, 16]. Furthermore, in overt GIB, procedures done within 5 days of the onset of bleeding or in patients who had required transfusions before VCE have been shown to have a higher pathology detection rate [14, 18]. We did not document either the duration of symptoms or any therapeutic interventions before VCE in this study.

The role of VCE in the evaluation of abdominal pain has been questioned because of a low diagnostic yield [6, 16]. The yield is higher in patients with abnormal biologic markers, erythrocyte sedimentation rate (ESR), and C-reactive protein (CRP) [14]. In our study, 71.4% of the patients with abdominal pain as their sole indication for VCE had normal findings on VCE (Supporting Table 1). We did not record biologic markers in this study. Given the relatively low pathology identification rate, we recommend that VCE should not be considered as a diagnostic tool in patients with the sole complaint of abdominal pain, especially in resource-limited environments.

Gastritis was the most common nonsmall bowel finding in 33.9% of the patients. Other diagnoses included duodenitis, colon ulcers, and melanosis coli. A similar detection rate of gastritis in 27% was reported in a study by Toy et al. [6]. It is unclear if the nonsmall bowel findings in our study were previously identified by the procedures done before referral for VCE, as they were not regularly recorded.

The most common finding in our study was angiodysplasia, in 19.4%. This is in keeping with most other reports that consistently identified this vascular anomaly as the most frequently detected pathology [16–18]. Concerning obscure (occult and overt) GIB, angiodysplasia was the most common finding in our study, detected in 35.1% of the patients. Previous studies have reported rates between 33% and 50% [6, 17, 18]. The frequency of detection of a mass/polyp in our study was about 7%, which was similar to one meta-analysis, including reports from mostly Asian and Western countries, which showed a prevalence of neoplastic lesions in VCE at 8.8% [17]. There are limited published data with a study population similar to ours for correlation. We did not follow up with patients in the present retrospective study after VCE to analyze the impact of the procedure, including whether histological diagnosis of the lesions was pursued.

Peculiar findings in our study compared with published literature were VCE findings of suspected intestinal TB and intestinal helminthiasis, which occurred in 5.6% and 2.8% of the patients, respectively. The SSA region has a huge burden of infectious diseases with protean GIT manifestations. The indications for VCE in the two patients with eventual findings of helminths were overt GIB with anemia in one and altered bowel habits in another. Similar to the three patients in our study with features of intestinal TB, cases presenting with overt GIB and eventual diagnosis of enteric TB have been reported from the region [19]. Patients who had lesions suspicious of gastrointestinal tuberculosis were referred for screening, and potential diagnosis, of other sites for microbiological diagnosis. Given the widespread availability of cheaper and convenient diagnostic alternatives for the above infections, it is unlikely that there will be reliance on VCE in the investigation of patients with these conditions.

As is standard of care with VCE, the captured recording was downloaded and subsequently reviewed and reported by the gastroenterologist. Drawbacks for such a setup include lack of multidimensional images, inability to manipulate the capsule, absence of interventional capability, and physician's fatigue in reading frames [20, 21]. These can be partially overcome by commercially available computer software that reduces the reading of frames by posting abnormal frames and by using suspected blood indicators [1]. In addition, manipulated transit and localization of lesions for image quality improvement can be achieved by magnetic-directed localization. Magnetic capsule endoscopy (MCE) deploys an external magnetic controller resulting in a reduction of transit time while enabling maneuvers on the capsule without patient discomfort [2]. Lastly, concerning image interpretation, the role of AI in the identification of pathology and optical biopsy is increasingly being recognized [4, 20, 21]. Convolutional neural network (CNN), a machine deep learning algorithm, has been studied in VCE with encouraging results of sensitivity and specificity of more than 90% in the detection of angiodysplasia [4, 5]. Although the use of AI shows great promise, the main drawback of this intelligence in SSA will be the costs associated with acquiring, mastering, and evaluating AI technology.

Our study adds to the available literature on the use and yield of VCE, with data from an area (SSA) not well studied previously. The high detection rates should impact clinical outcomes by influencing decisions on therapeutic interventions. Our study had a few limitations: first, all reports were from a single gastroenterologist and, thus, are subject to reporter bias. However, this has also been the case in other studies. In addition, we did not evaluate the impact and follow-up of VCE results on patient management since this was out of the scope of this study. Our results are from a single center and, thus, may not be generalizable. Lastly, we did not perform a cost analysis of VCE, which is a vital factor that public and private health managers consider before VCE can be integrated into regular gastroenterological practice in the region.

## 5. Conclusion

The use of VCE in a single center in Kenya reveals a high diagnostic yield, identifying obscure gastrointestinal bleed as the most common indication and small bowel tumors and angiodysplasia as the most common pathologies visualized. The findings are also largely consistent with previous studies from other parts of the world.

## Figures and Tables

**Table 1:** Investigations before capsule endoscopy.

Procedure	(Number = *N*, %)
Upper GI endoscopy	*N* = 72/72, 100%
Lower GI endoscopy	*N* = 69/72, 95.8%
CT abdomen	*N* = 29/72, 40.3%
Angiography	*N* = 4/72, 5.6%
Barium swallow	*N* = 2/72, 2.8%
Enteroscopy	*N* = 1/72, 1.4%

**Table 2:** Abnormal findings of video capsule endoscopy.

Small bowel abnormalities (*N* = 72, %)	
Angiodysplasia	14 (19.4%)
Duodenitis	6 (8.3%)
Suspected Crohn's disease	5 (6.9%)
Mass/polyps	5 (6.9%)
Suspected intestinal tuberculosis (TB)	4 (5.6%)
Celiac disease	3 (4.2%)
Ileitis	2 (2.8%)
Helminths	2 (2.8%)
Blood without definite lesions	1 (1.4%)
Bile diarrhea	1 (1.4%)
Lymphoma	1 (1.4%)

**Nonsmall bowel abnormalities (*N* = 72, %)**	

Gastritis	19 (26.4%)
Colon ulcers	2 (2.8%)
Melanosis coli	1 (1.4%)

**Table 3:** Small bowel findings based on indications.

Endoscopic findings only in small bowel	Indication									
	Occult GIB *N* = 17	Overt GIB *N* = 20	Abdominal pain *N* = 18	Anemia *N* = 11	Altered bowel habits *N* = 17	Crohn's disease *N* = 5	Weight loss *N* = 7	Miscellaneous		Overall *N* = 72
								Elevated chromogranin A *N* = 1	GIST tumor treatment *N* = 1	
Angiodysplasia, *n* (%)	6 (35.3)	7 (35.0)	2 (11.1)	3 (27.3)	2 (11.8)	0 (0.0)	0 (0.0)	0 (0.0)	1 (100.0)	14 (19.4)
Mass/polyp, *n* (%)	0 (0.0)	2 (10.0)	1 (5.6)	0 (0.0)	2 (11.8)	0 (0.0)	1 (14.3)	1 (100.0)	0 (0.0)	5 (6.9)
Crohn's disease, *n* (%)	0 (0.0)	0 (0.0)	2 (11.1)	0 (0.0)	2 (11.8)	2 (40.0)	1 (14.3)	0 (0.0)	0 (0.0)	5 (6.9)
Intestinal TB, *n* (%)	1 (5.9)	3 (15.0)	0 (0.0)	1 (9.1)	0 (0.0)	0 (0.0)	0 (0.0)	0 (0.0)	0 (0.0)	4 (5.6)
Duodenitis, *n* (%)	1 (5.9)	2 (10.0)	2 (11.1)	0 (0.0)	1 (5.9)	2 (40.0)	0 (0.0)	0 (0.0)	0 (0.0)	6 (8.3)
Blood without definite lesion, *n* (%)	0 (0.0)	1 (5.0)	0 (0.0)	0 (0.0)	0 (0.0)	0 (0.0)	0 (0.0)	0 (0.0)	0 (0.0)	1 (1.4)
Helminths, *n* (%)	1 (5.9)	0 (0.0)	0 (0.0)	1 (9.1)	1 (5.9)	0 (0.0)	0 (0.0)	0 (0.0)	0 (0.0)	2 (2.8)
Bile diarrhea, *n* (%)	0 (0.0)	0 (0.0)	1 (5.6)	0 (0.0)	1 (5.9)	0 (0.0)	0 (0.0)	0 (0.0)	0 (0.0)	1 (1.4)
Celiac disease, *n* (%)	1 (5.9)	0 (0.0)	1 (5.6)	1 (9.1)	2 (11.8)	0 (0.0)	0 (0.0)	0 (0.0)	0 (0.0)	3 (4.2)
Ileitis, *n* (%)	1 (5.9)	0 (0.0)	0 (0.0)	1 (9.1)	0 (0.0)	0 (0.0)	0 (0.0)	0 (0.0)	0 (0.0)	2 (2.8)
Lymphoma, *n* (%)	0 (0.0)	1 (5.0)	0 (0.0)	0 (0.0)	0 (0.0)	0 (0.0)	0 (0.0)	0 (0.0)	0 (0.0)	1 (1.4)

## Data Availability

The data that support the finding of this study are included within the article.

## Supporting Information

Supporting Table 1: Findings in patients with abdominal pain as sole indication for VCE. Supporting Figure 1: Comparison of VCE findings in patients older than 60 years and those younger than 60 years.
