# Supplementary material for: Indications and Outcomes of Video Capsule Endoscopy in Sub-Saharan Africa: A 5-Year Single-Center Experience in Nairobi, Kenya
Source: J Trop Med. 2025 Jul 23;2025:6495299. doi: 10.1155/jotm/6495299 (PMC12310308; doi:10.1155/jotm/6495299)

**Supplementary Material**

**Supplementary Table 1**. Findings in patients with abdominal pain as sole indication for VCE

|  | Abdominal pain as the sole positive indication for VCE |
| --- | --- |
|  |  |
| Indications | N=7 |
| Angiodysplasia, n(%) | 0 (0.0) |
| Mass/ Polyp, n(%) | 0 (0.0) |
| Ileo-jejunitis, n(%) | 0 (0.0) |
| Crohn's disease, n(%) | 0 (0.0) |
| Intestinal TB, n(%) | 0 (0.0) |
| NSAID enteropathy, n(%) | 0 (0.0) |
| Non specific ulcerations, n(%) | 0 (0.0) |
| Duodenitis, n(%) | 1 (14.0) |
| Blood without definitive lesion, n(%) | 0 (0.0) |
| Helminthiasis, n(%) | 0 (0.0) |
| Miscellaneous, n(%) | 1 (14.0) |
| Other findings, n(%) |  |
| Bile diarrhea | 0 (0.0) |
| Celiac disease | 0 (0.0) |
| Colon ulcers | 0 (0.0) |
| Ileitis | 0 (0.0) |
| Ileitis (infective) | 0 (0.0) |
| Lymphoma | 0 (0.0) |
| lymphoma; melanosis coli | 0 (0.0) |
| Ulcerative colitis (colon ulcers) | 1 (14.0) |

**Supplementary Figure 1.** Comparison of VCE findings in patients older than 60 years and those younger than 60 years


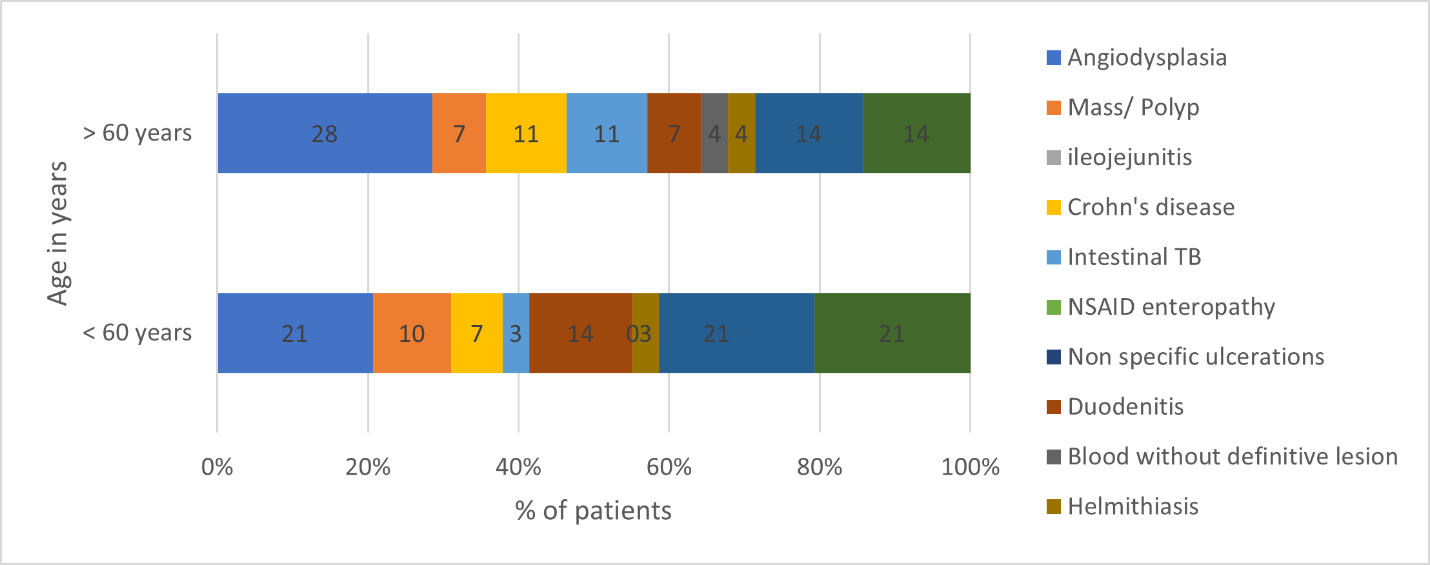

Supplement: Supporting Information — Additional supporting information can be found online in the Supporting Information section. [file 6495299.f1.docx]
